# Patient Reported Outcome Measures in Dysphagia Research Following Stroke: A Scoping Review and Qualitative Analysis

**DOI:** 10.1007/s00455-022-10448-y

**Published:** 2022-04-25

**Authors:** Jennifer Moloney, Julie Regan, Margaret Walshe

**Affiliations:** Department of Clinical Speech and Language Studies, Trinity College Dublin, 7-9 South Leinster Street, Dublin 2, Ireland

**Keywords:** Quality of life, Dysphagia, Stroke, Patient-reported outcomes, Scoping review

## Abstract

Patient reported outcome measures (PROMs) are commonly used to evaluate the impact of a health condition on quality of life (QOL). This study aimed to identify the range of PROMs that are currently in common use in clinical trials in dysphagia following stroke and to qualitatively analyse these PROMs by mapping the content to both the International Classification of Functioning and Disability Framework (ICF) and the Core Outcome Measures in Effectiveness Trials (COMET) Taxonomy for outcome classification. With consideration for the PRISMA-ScR checklist, a scoping review was conducted to identify commonly used PROMs in randomised controlled trials reported in persons with dysphagia stroke. A search of five databases was conducted. Studies were excluded if they included pediatric participants i.e. < 18 years of age, or if the text was not available in the English language. 110 papers met the inclusionary criteria. Twelve of these 110 papers included a dysphagia PROM. Two PROMs were identified as being in common use—the SWAL-QOL and the EAT-10. These two tools consisted of 47 items and 78 meaningful concepts, which were subsequently mapped to the ICF and the COMET Taxonomy. Mapping to the ICF showed that neither tool directly assessed the impact of ‘Environmental Factors’ on the experience of dysphagia. Mapping to the COMET Taxonomy showed that neither tool considered the impact of ‘Role Functioning’ on the person’s experience of dysphagia. The development of a suitable and appropriate patient-reported assessment tool for use in those with dysphagia following stroke is warranted.

**Supplementary Information:**

The online version contains supplementary material available at 10.1007/s00455-022-10448-y.

## Introduction

Patient reported outcome measures (PROMs) have been used for many years to gain insights into a patient’s view of their physical symptoms, their functional abilities and their perception of their overall psychosocial well-being in relation to their health status [1, 2]. As a result, in both research and clinical practice, an assessment of a person’s quality of life (QOL) and psychosocial well-being is most often made using these questionnaire based tools [3, 4]. The term ‘patient- reported’ is included in the description of these outcome measures to denote one important feature—the tool is used to allow the person to make a self-evaluation or assessment of their own satisfaction, well-being and happiness [5, 6].

In persons with dysphagia, PROMs are widely used to explore the impact of dysphagia on QOL and to evaluate the effectiveness of treatment and intervention approaches. At present, there are upwards of 30 tools currently published and available in the literature [7]. A key consideration for any clinician or researcher in the selection of which PROM to use, is the quality of the tool. This judgement is most often made with consideration for its underlying psychometric properties, including its reliability and appropriateness for use within a given clinical population. However, another important consideration in the selection of an appropriate PROM is the content and focus of the tools themselves i.e. what aspects of the person’s experience do the items in the tool consider and assess [8, 9]. Unless a PROM truly and comprehensively incorporates the perspective of the person or clinical population for which it is intended, then it is unlikely to differ significantly from traditional measurement tools [10].

The International Classification of Functioning and Disability Framework (ICF) has been successfully used in previous studies to evaluate the comprehensiveness and suitability of the content of outcome measures that are used in both healthcare research and clinical practice [11–14]. The ICF framework can be used to describe a person’s experience of health by considering four key components—Body Functions, Body Structure, Activities and Participation and Environmental Factors. By mapping the content of selected PROMs to the various components and categories within the ICF, an evaluation of the scope and breadth of a tool or outcome measure can be made [15].

In more recent years, there has also been a move towards the development of core outcome sets (COS) in healthcare research. A COS recommends through consensus, the minimum set of outcomes that should be considered and measured in research concerned with specific conditions and populations [16]. In an attempt to support this movement, the Core Outcome Measures in Effectiveness Trials (COMET) initiative developed a detailed taxonomy that can be used in the analysis and classification of specific outcome measures—The COMET Taxonomy [17]. This taxonomy comprises four core areas and 38 different outcome domains. Similar to studies that have used the ICF, by mapping a given PROM to the COMET Taxonomy, potential gaps and limitations in the content of a specific tool or outcome measure can be highlighted [18].

Ongoing research has demonstrated the significant impact that dysphagia can and does have on psychosocial well-being and QOL in persons with dysphagia following stroke [19, 20]. Furthermore, the need for healthcare clinicians to be aware of and address this important aspect of dysphagia care has been acknowledged in a number of a stroke clinical care guidelines [21, 22]. At present, the research team does not know of any PROM that has been specifically developed for use in persons with dysphagia following stroke. As a result, both researchers and clinicians are limited to using a PROM that has been developed with a generic dysphagia population in mind and that may or may not have been validated for use with those who have had a stroke. Therefore, the content assessed by these tools may not be adequately capturing the wide-ranging and complex experiences reported by this clinical group [19, 20].

This study aims to identify the range of PROMs that are currently in common use in clinical trials in dysphagia research following stroke. Once identified, it is likely that the psychometric properties of these PROMs will have been previously addressed and reviewed in the literature [7, 23]. Therefore, this study also aims to qualitatively analyse the content of these tools using both the ICF framework and the COMET Taxonomy. In doing so, the appropriateness and relevance of the information that is gathered and evaluated by these tools can then be considered.

## Methods—Stage 1: Scoping Review

The first stage of this study involved conducting a scoping review to identify and select PROMs that are commonly used in dysphagia randomised controlled trials (RCTs) following stroke. This scoping review was guided by the Preferred Reporting Items for Systematic Review and Meta-Analyses Extension for Scoping Reviews (PRISMA-ScR) Checklist [24] and the methodological framework outlined by Arskey and O’Malley in 2005 [25]. The protocol for the scoping review was prospectively published in November 2020, on the COMET database and can be accessed at comet-initiative.org/Studies/Details/1748.

### Eligible Studies

Published studies were included if they reported a RCT, which examined the effectiveness of a named dysphagia intervention in adults following stroke. For the purpose of this study, a RCT was defined as ‘a trial in which subjects are randomly assigned to one of two groups: one (the experimental group) receiving the intervention being tested, and the other (comparison or control) receiving an alternative (conventional) treatment’ [26, p. 164]. Furthermore, the study had to contain a singular measure of swallowing performance or swallowing related outcomes.

Studies were excluded if they included pediatric participants i.e. < 18 years of age, or if the text was not available in the English language. Where conference abstracts were deemed to report on the same data as a published original paper, the published paper was given preference and the abstract was counted as a duplicate.

### Search Strategy

A comprehensive electronic search strategy was developed including terms that were thematically related to stroke, dysphagia and randomised controlled trial. The search was completed on December 13th 2020. Five databases were searched from inception to this date: CINAHL, The Cochrane Database, EMBASE, Web of Science and PubMed. (See Supplementary Material).

### Data Extraction

All studies identified following the electronic search were uploaded to the online Covidence platform (www.covidence.org) to support the screening and selection process. Using this platform, the title, abstract and full-text of relevant studies was reviewed independently by two members of the research team. Any discrepancies that arose were discussed in detail and a consensus was reached regarding their selection.

Once study review and screening was completed, the relevant data was extracted and inputted onto a data extraction sheet which was developed on Excel. On this data extraction sheet, the details of each study were listed alphabetically, alongside their basic characteristics (i.e. year published, intervention) and the specific outcome measures that were used. The outcome measures referenced across the selected studies were then extracted and listed and duplicates were removed. The remaining list of outcome measures was screened and those measures which did not meet the relevant inclusion criteria were excluded from further analysis.

The initial screening phase was completed to ensure that the outcome measure was relevant to persons who have had a stroke. Therefore, the outcome measures were only accepted if they:

- Directly evaluated swallowing or
- Directly evaluated stroke but had at least one item that evaluated swallowing.

Following the initial screening phase, the list of outcome measures were reviewed once again to ensure that the measure could be deemed to be a PROM. In order to achieve this, the following additional criterion was applied:

- The measure had to have at least one item that sought feedback or information directly from the person with dysphagia.

The final list of identified PROMs was then reviewed and screened one last time. The purpose of this final screening phase was to ensure that the PROMs could be deemed to be in ‘common’ use, and that they were capable of providing some level of meaningful data. Similar to other studies, which have explored the content of outcome measures [11, 14], this was achieved by applying two final criteria. The remaining PROMs were only accepted if:

- They were used by at least two different author groups (signifying its use as a common outcome measure) and
- If there was at least some published data available to support their psychometric properties.

## Methods—Stage 2: Qualitative Analysis of Content

The second stage of this study involved the mapping of each identified PROM to both the ICF and the COMET Taxonomy for Outcome Measures. This mapping exercise was completed independently by two of the authors. The senior author has been working with ICF classifications since 2003, and is actively involved in other research associated with the COMET taxonomy. Furthermore, two of the three researchers completed online training with the COMET taxonomy initiative.

### ICF Mapping Process

In order to complete the ICF mapping process, individual measurement items or domains within each PROM were listed and considered individually. The key concepts in each item/domain were then extracted and mapped to the most suitable ICF category. This mapping exercise was guided by previously used and well-established coding rules [9, 27]. A summary of these coding rules is available in Table 1.

**Table 1:** ICF coding rules

Number	Rule
1	Become familiar with the structure and concepts within the ICF prior to completing a mapping exercise
2	Link each item to the most precise ICF category
3	If the content of a concept is not explicitly named in the ICF category, document this content separately
4	Do not use the ‘unspecified’ categories in the ICF, instead link the concept to the related higher level category
5	If there is not enough information regarding the concept to link it to an ICF category, document the concept as being ‘not defined’
6	If the concept is not contained in the ICF, but is clearly a personal factor, document the concept as ‘personal factor’
7	If the concept is not contained in the ICF but is not a personal factor, the concept should be documented as ‘not covered’
8	If the concept refers to a diagnosis of specific health condition, document the concept as ‘health condition’

By way of an example, the Dysphagia Handicap Index (DHI) [28] is a 25 item patient-reported questionnaire. If the DHI was being mapped to the ICF, each of the 25 questions would be treated as an individual item. Each item would then be considered separately and the relevant concepts in the item extracted. For item three on the DHI—‘I’m embarrassed to eat in public’—the extracted concepts would be ‘embarrassment’ and ‘eating in public’. These two concepts would then linked to the most relevant categories within the ICF. ‘Embarrassment’ would be linked to ‘e460—Societal attitudes’, which is listed within the Environmental Factors component of the framework. By comparison ‘eating in public’ would be linked to ‘d920—Recreation and leisure’, which is listed within the Activities and Participation component.

The ICF mapping process was completed by the lead researcher and was independently reviewed by the other members of the research team. Any disagreements were discussed and a consensus decision was reached.

### COMET Taxonomy Mapping Process

In mapping the identified PROMs to the COMET Taxonomy, each item or domain within the PROM was again considered individually. The principal concept within each item was determined and this concept was then mapped to the taxonomy. The descriptive information provided for each core area and outcome domain within the COMET Taxonomy was used to guide the mapping process.

Again, using the same example from the ICF Mapping Process, the third item on the DHI [28]—‘I’m embarrassed to eat in public’—would be mapped to the core area ‘Life Impact’. Within this core area, the item would be further mapped to ‘Social functioning’. By way of comparison, item number 11 on the DHI, ‘I eat less because of my swallowing problem’, would be mapped to the core area ‘Physiological/Clinical’ and the outcome domain ‘Metabolism and nutrition outcomes’.

The COMET Taxonomy mapping process was completed by the lead researcher and was reviewed independently by the research team. Any disagreements were discussed and a consensus decision was reached.

## Results—Stage 1: Scoping Review

### Study Selection

**Fig. 1:**
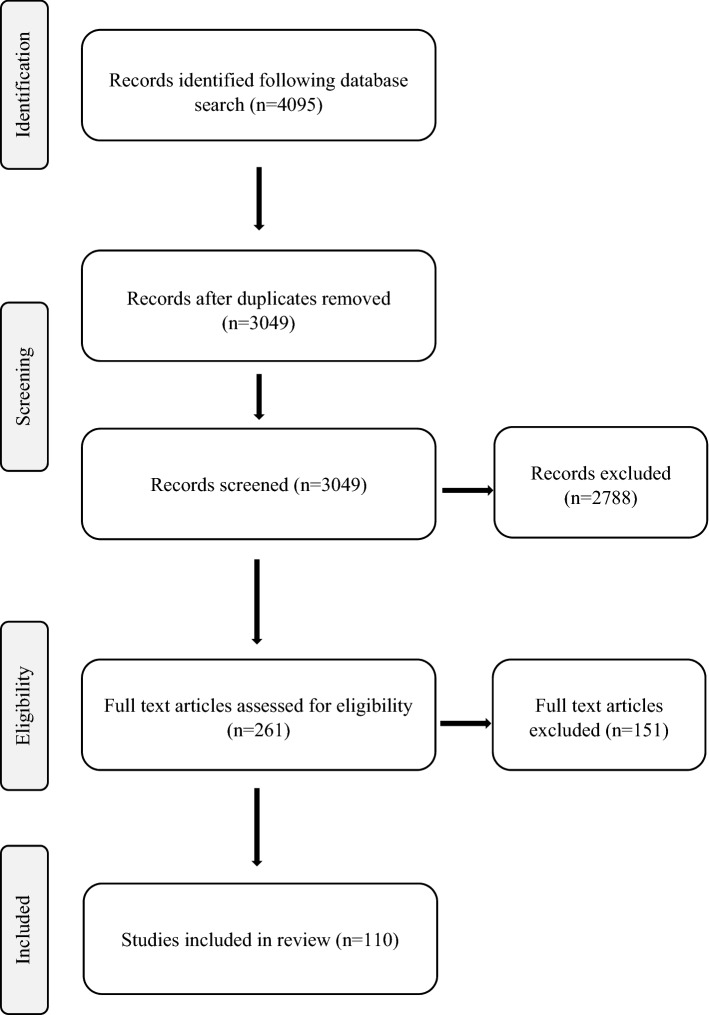
PRISMA flow diagram

Electronic database searching resulted in the identification of 4095 articles. Following removal of duplicates, 3049 articles were screened for inclusion. Following title and abstract screening, 2788 articles were removed, leaving 261 articles to be assessed for eligibility. 151 more articles were excluded following full text review, leaving 110 articles which were included in the final review (Fig. 1).

### Outcome Measure Selection

From the 110 articles that were included in the final review, 30 distinct outcome measures were identified following the initial screening process. Each of these outcome measures either (a) directly evaluated swallowing or (b) directly evaluated stroke outcomes but had at least one item that was related to swallowing.

On further screening, 22 of these outcome measures were subsequently excluded, as they did not contain at least one item which sought feedback or information directly from the person with dysphagia i.e. they were not classified as a PROM. Of the remaining eight outcome measures, six were excluded, as a) at least two groups of authors did not cite them, or b) there was no published data available to support their psychometric properties.

This resulted in the selection of two PROMs—the Swallowing Quality of Life Questionnaire (SWAL-QOL) [29] and the Eating Assessment Tool (EAT-10) [30]. The SWAL-QOL was cited as an outcome measure in ten separate clinical trials, while the EAT-10 was cited in two clinical trials. Therefore, 12 of the 110 articles that were screened included a commonly used PROM to evaluate the effectiveness of the intervention under investigation (Table 2).

**Table 2:** Results of outcome measure screening process

Outcome measure and no. of times cited in articles	Seeks feedback from person with dysphagia	Cited by at least two author groups	Published psycho-metric data available
Clinical Dysphagia Scale (*n* = 1)	✗	–	–
Cough Reflex Grading Score (*n* = 1)	✗	–	–
Dysphagia Handicap Index (DHI) (*n* = 1)	✓	–	–
Dysphagia Severity Rating Scale (*n* = 6)	✗	–	–
*Eating Assessment Tool (EAT-10) (n = 2)*	✓	✓	✓
EuroQOL-5 Dimension (EQ-5D) (*n* = 1)	✓	–	–
Functional Oral Intake Scale (FOIS) (*n* = 16)	✗	–	–
Generic QOL Inventory (GQOL-74) (*n* = 1)	✓	–	–
Kubota Toshio Swallow Test (*n* = 4)	✗	–	–
Lip Force Test (*n* = 1)	✗	–	–
Mann Assessment of Swallowing Ability (MASA) (*n* = 5)	✗	–	–
Modified Mann Assessment of Swallowing Ability (MMASA) (*n* = 2)	✗	–	–
Numerical Rating Self-Report Scale (*n* = 1)	✓	–	–
Penetration Aspiration Scale (PAS) (*n* = 30)	✗	–	–
Patient Self-Perception Score (*n* = 1)	✓	–	–
Parramatta Hospitals’ Assessment of Dysphagia (PHAD) (*n* = 1)	✗	–	–
Repetitive Saliva Swallowing Test (*n* = 2)	✗	–	–
Royal Brisbane Hospital Outcome Measure for Swallowing (RBHOMS) (*n* = 2)	✗	–	–
Standardised Swallowing Assessment (SSA) (n = 10)	✗	–	–
Swallow Function Scoring System (*n* = 2)	✗	–	–
*Swallowing Quality of Life Questionnaire (SWAL-QOL) (n = 10)*	✓	✓	✓
Timed Water Swallow Test (*n* = 1)	✗	–	–
Videofluoroscopic Dysphagia Scale (VDS) (*n* = 8)	✗	–	–
Visual Analogue Scale (*n* = 1)	✓	–	–
Visual Analogue Satisfaction Scale (*n* = 1)	✗	–	–
Volume Viscosity Swallow Test (*n* = 1)	✗	–	–
Water Drinking Test (*n* = 1)	✗	–	–
Water Intake Test Score (*n* = 1)	✗	–	–
Water Swallow Test (*n* = 6)	✗	–	–
Watian Drinking Water Test (*n* = 1)	✗	–	–

## Results—Stage 2: Qualitative Analysis of Content

### ICF Mapping Process

The two identified PROMs that were included in the qualitative analysis, consisted of 54 individual items that required conceptualisation and mapping. On review, seven of these items were subsequently excluded as they did not relate directly to swallowing (e.g. SWAL-QOL Item 43—‘In the last month how often have you had trouble staying asleep’). These items were therefore not included in the identification and mapping of relevant concepts. (See Supplementary Material). The remaining 47 items were independently mapped to the ICF by two of the authors with an inter-rater agreement of 100%.

Consideration of the remaining 47 items resulted in the identification of 85 individual concepts. Seven of the 85 concepts were subsequently excluded as they could not be directly linked to the ICF. These concepts included factors relevant to swallowing such as ‘frustration’, ‘caution’ and ‘apathy’, but as they are not currently classified under the ICF, they could not be included.

The remaining 78 concepts were mapped  to 14 different ICF categories. 36% (*n* = 5) of these concepts related to Body Functions, 7% (*n* = 1) related to Body Structures and 57% (*n* = 8) related to Activity and Participation. No concepts in either PROM were deemed to be related to Environmental Factors. The most commonly identified ICF category across both PROMs was b510—Ingestion Functions, with 35% (*n* = 27) of concepts related directly to this category.

The SWAL-QOL represented 53 of the 78 concepts that were mapped to the ICF. The majority of these concepts (*n* = 32, 60%) were related to Body Functions i.e. the physiological functions of the body systems. The remaining concepts (*n* = 21, 40%) were related to Activities and Participation i.e. the person’s ability to complete a task and/or be involved in a life situation.

By comparison, the EAT-10 represented a more even spread of concepts across the ICF, with 52% (*n* = 13) of concepts related to Body Functions and 44% (*n* = 11) related to Activities and Participation. The EAT-10 contained one concept that was mapped to Body Structures i.e. the anatomical parts of the body.

### COMET taxonomy mapping process

As previously outlined, the two PROMs included in the qualitative analysis consisted of 54 individual items. Again, seven of these items were excluded as they did not relate directly to swallowing, meaning that 47 items were included in the mapping process. (See Supplementary Material).

Of these 47 items, 27 mapped to the core area ‘Life Impact’. Within this area, the most commonly mapped domain was ‘Emotional functioning/wellbeing’. The other 20 items mapped to the core area’ Physiological/Clinical’. Within this area, the most commonly mapped domain was ‘Gastrointestinal outcomes’.

The SWAL-QOL represented 37 of the items that were mapped to the COMET Taxonomy. Of these 37 items, 57% (*n* = 21) were mapped to the core area ‘Life Impact’ with the most common outcome domain within this core area being ‘Emotional functioning/wellbeing’ (*n* = 13, 62%). The remaining 16 items were mapped to the core area ‘Physical/Clinical’ with ‘Gastrointestinal outcomes’ being the most common outcome domain mapped within this area (*n* = 9, 56%).

The EAT-10 represented the other 10 items that were mapped to the COMET Taxonomy. Of these 10 items, 60% (*n* = 6) were mapped to the core area ‘Life Impact’ and 40% (*n* = 4) were mapped to the core area ‘Physical/Clinical’. The items within the EAT-10 demonstrated a relatively even spread across the outcome domains that were mapped.

## Discussion

The results of this study demonstrate the limited inclusion of PROMs to evaluate patient perspectives and priorities in dysphagia intervention research following stroke. Of the 110 studies included in this review, just over 10% (*n* = 12) included a commonly used and validated PROM. Indeed, only 17% (*n* = 19) of studies included any measure of patient perception, regardless of validity, reliability or common use. Of the 12 studies that did include a PROM, the two measures that were used in these studies—the SWAL-QOL [30] and the EAT-10 [31]—are both generic dysphagia assessment tools that were developed with a general dysphagia population in mind.

The SWAL-QOL was cited in 10 of the 12 RCTs which included a PROM. This may not be surprising, given the sound psychometric properties reported in the literature and the validation of its use in many clinical groups with dysphagia [7, 31]. However, the feasibility of administering the SWAL-QOL has long been questioned in the literature, given that the person with dysphagia must make a rating on a 5 point scale and different instructions are given for different sections throughout the tool. As a result, the accessibility of the tool may prove particularly difficult for persons with cognitive and/or communication challenges [32, 33]. This issue becomes especially relevant in individuals presenting with dysphagia following stroke, given the wide range of concomitant impairments, including aphasia, which may present in this group [34, 35].

By comparison, the EAT-10 may be relatively quick and easy to administer, with only 10 items which the person with dysphagia is required to rate on a scale of 0 to 4 [24]. Alongside its use as a PROM, numerous studies have also shown the ability of the EAT-10 to be used a screening tool to detect dysphagia and aspiration in some clinical groups [36–38]. However, the psychometric properties of the EAT-10 have recently been questioned in the literature [39, 40]. Furthermore, it has been suggested that the EAT-10 may lack sufficient depth and detail to track and demonstrate changes in the person’s perception of their swallowing problem [40]. The authors of the tool acknowledge that in order to achieve ease in administration and use, it was necessary to limit the depth and breadth of the items that were assessed [30]. As a result, items that specifically assess the social, emotional and functional impact of dysphagia were omitted; meaning the very use of the EAT-10 in the assessment of psychosocial well-being and QOL is questionable.

Beyond the use and feasibility of the two PROMs that were highlighted following the scoping review, the qualitative analysis of these PROMs also suggested some important findings. When the identified concepts within the SWAL-QOL were mapped to the ICF, the majority of items were related to Body Functions, suggesting that the outcome measure focuses mainly on the physiological aspects of the swallow process. However, all of the other concepts within the measure were related to the Activities and Participation chapter within the ICF framework. This suggests that the measure also recognises the restrictions that dysphagia can place on a person’s involvement in everyday activities and day-to-day life situations.

A notable gap within the SWAL-QOL however, was the lack of consideration for Environmental Factors. Within Environmental Factors, the ICF considers the impact of important concepts such as relationships and support structures, familial and societal attitudes, social policy and health service delivery. It is widely recognised that a significant link exists between dysphagia and social isolation [41]. Furthermore, previous research has demonstrated the significant impact that ‘Environmental Factors’ has on the experiences of both persons living with long-term dysphagia following stroke and those living with dysphagia during the stroke rehabilitation journey [19, 20]. The absence of any item that can be linked to the Environmental Factors chapter of the ICF, suggests that the SWAL-QOL may not be holistic enough to consider the complex and wide-ranging impact that dysphagia can have in this population.

A similar gap in content was evident when the EAT-10 was mapped to the ICF. Again, the items in this measurement tool represented categories in both the Body Functions and Activities and Participation chapters, and also included one concept that was mapped to Body Structures. However, the lack of consideration for and inclusion of items that could be linked to Environmental Factors was noteworthy, with no item in the EAT-10 mapped directly to this chapter of the ICF.

When both the SWAL-QOL and the EAT-10 were mapped to the COMET Taxonomy, only two of a possible five core areas were represented—Physiological/Clinical and Life Impact. However, given that these are PROMs, this is to be expected, as the other core areas within the COMET Taxonomy—Death, Resource Use and Adverse Events—are not suitable to be rated by the person with dysphagia. Of note, within the core area of Life Impact, the outcome domain ‘Role functioning’ was only mapped twice—both times within the SWAL-QOL. Again, this suggests that both of these outcome measures may not be giving enough consideration to the impact that dysphagia can have on a person’s role and function within society.

There are known limitations to mapping outcome measures to the ICF. Firstly, a number of concepts may not be classified under the current version of the framework and so may need to be excluded [14]. In order to mitigate the impact of this on the findings of the study, a second framework was also used—the COMET Taxonomy. The use of both frameworks in the mapping exercise ensured that all relevant assessment items were accounted for and also offered a level of data triangulation. Similar gaps in the identified PROMs were highlighted when mapped to both frameworks and so this strengthens this finding.

Secondly, limitations in the inter-rater reliability of mapping outcome measures to the ICF have been recognised in the literature [42, 43]. A certain level of subjectivity and interpretation is required in the development of concepts from assessment items. Further subjectivity and interpretation can exist in linking these concepts to a relevant ICF chapter and category. For example, when considering item number 5 in the EAT-10—‘Swallowing pills takes extra effort’—the concept of pills was mapped to the Activities and Participation component, the ‘Self-care’ chapter and the category ‘Looking after one’s health’, as it was interpreted that pills in this context was related to the activity of taking medication and the person’s ability to do this. However, if the concept of pills had been taken in isolation, without consideration for the context in which the concept was phrased, it may have be mapped as ‘Products or substances for personal consumption’ under the ‘Environmental Factors’ component of the framework. Similar biases and nuances in interpretation are likely to have occurred in mapping the tools to the COMET Taxonomy outcome domains.

In order to minimise the impact of bias and interpretation, the study would have been strengthened if the entire mapping process had been completed independently by a number of researchers and disagreements discussed until consensus reached. If this approach was taken, the reliability of the mapping process could also have been evaluated statistically by computing percentage exact agreement scores, for example. However, the timeline and resources for completion of the study restricted what was possible.

Finally, it should be noted that limiting the inclusion criteria to include only RCTs will have restricted the number of PROMs that were subsequently included in the qualitative analysis. Furthermore, it was noted that many of the published RCTs in dysphagia following stroke have been completed in large centres based predominantly in the United States and China, with this bias potentially limiting the PROMs that were included. However, as the primary focus of a COS is to establish the minimum outcomes that should be measured and reported in all clinical trials on a specific condition, the authors felt it was appropriate to only include RCTs.

Notwithstanding these limitations, the findings of this study suggest that the development of a stroke-specific dysphagia PROM may be necessary. Such a measurement tool should include a broad range of assessment items that equally target Body Functions, Activity and Participation and Environmental Factors, while adhering to the Consensus-based Standards for the selection of Health Measurement Instruments (COSMIN) checklist [44]. With consideration for previous literature which has explored the experiences of persons with dysphagia following stroke [19, 20] factors to include within the environmental domain might include consideration of both formal and informal supports and if these supports are meeting the needs of the person with dysphagia, or the person’s level of satisfaction with the availability and relevance of dysphagia healthcare services. Furthermore, any PROM should ideally be developed in collaboration with the clinical population for which the measure is intended to be used, otherwise the relevance and appropriateness of the tool cannot be guaranteed [45].

It is acknowledged that this study has not considered and mapped all the possible PROMs that are available and in use for persons with dysphagia following stroke (e.g. the DHI), as these were not commonly used in the included studies in this review. However, similar generic dysphagia QOL tools that are available, will also likely to be limited in their ability to truly capture and measure the complex perspectives and experiences [46]. Consequently, it has been suggested that disease-specific PROMs are necessary for use in clinical trials following stroke, where the intervention goals include reduction in symptoms, improvement in function or a change in QOL [46]. The need for a disease-specific dysphagia QOL assessment tool to support day to day clinical practice has also been reported by clinicians working internationally with people with dysphagia following stroke [47].

Finally, it is worth noting that the findings of this study suggest that dysphagia research following stroke is currently lacking a uniform and consistent approach to outcome measurement in general. A total of 30 outcome measures were identified in the first stage of the scoping review. Beyond the Penetration Aspiration Scale [48], which was cited 30 times, there was significant heterogeneity in the other measures that were included. Consideration should therefore be given to the development of a COS for dysphagia research following stroke, with this core outcome set including a stroke-specific dysphagia PROM.

## Conclusion

This study included a scoping review and qualitative analysis that identified and evaluated two PROMs that are currently in common use in dysphagia clinical trials following stroke—the SWAL-QOL and the EAT-10. The findings of this study highlight the lack of priority that current research gives to the evaluation and measurement of psychosocial well-being and QOL in dysphagia intervention trials in stroke. The findings also expose the lack of a stroke-specific dysphagia PROM, which comprehensively assesses the wide-ranging and broad experiences of individuals with dysphagia following stroke. Although a number of generic dysphagia QOL assessment tools are widely used in both research and clinical practice, the development of a suitable and appropriate PROM specifically for use in people with dysphagia following stroke may be warranted.

## Supplementary Information

Below is the link to the electronic supplementary material.

Supplementary file1 (DOCX 14 kb)

Supplementary file2 (DOCX 28 kb)

Supplementary file3 (DOCX 33 kb)
