# Correction to: Patient Reported Outcome Measures in Dysphagia Research Following Stroke: A Scoping Review and Qualitative Analysis

**DOI:** 10.1007/s00455-022-10507-4

**Published:** 2022-08-10

**Authors:** Jennifer Moloney, Julie Regan, Margaret Walshe

**Affiliations:** Department of Clinical Speech and Language Studies, Trinity College Dublin, 7-9 South Leinster Street, Dublin 2, Ireland

## Correction to: Dysphagia 10.1007/s00455-022-10448-y

The original version of the article unfortunately contained a mistake in the sequence of the second and third authors.

The incorrect authors name order is: Jennifer Moloney · Margaret Walshe · Julie Regan

The correct authors name order is: Jennifer Moloney · Julie Regan · Margaret Walshe

The author group has been updated above and the original article has been corrected.

