# Supplementary material for: Patient Reported Outcome Measures in Dysphagia Research Following Stroke: A Scoping Review and Qualitative Analysis
Source: Dysphagia. 2022 Apr 25;38(1):181–90. doi: 10.1007/s00455-022-10448-y (PMC9873730; doi:10.1007/s00455-022-10448-y)
Supplement: Supplementary file 1 — Supplementary file1 (DOCX 14 kb) [file 455_2022_10448_MOESM1_ESM.docx]

**Supplementary Material: Sample Search Strategy**

CINAHL Search Strategy

1. S1 (MH "Cerebrovascular Disorders") OR (MH "Basal Ganglia Cerebrovascular Disease+") OR (MH "Carotid Artery Diseases+") OR (MH "Cerebral Ischemia+") OR (MH "Cerebral Vasospasm") OR (MH "Intracranial Arterial Diseases+") OR ( (MH "Intracranial Embolism and Thrombosis") ) OR (MH "Intracranial Hemorrhage+") OR (MH "Stroke") OR (MH "Vertebral Artery Dissections") OR (MH "Stroke Patients") OR (MH "Stroke Units")
2. S2 TI ( stroke or poststroke or post-stroke or cerebrovasc* or brain vasc* or cerebral vasc or cva or apoplex ) or AB ( stroke or poststroke or post-stroke or cerebrovasc* or brain vasc* or cerebral vasc or cva or apoplex )
3. S3 TI ((brain or cerebr* or cerebell* or vertebrobasil* or hemispher* or intracran* or intracerebral or infratentorial or supratentorial or middle cerebral artery or MCA* or anterior circulation or posterior circulation or basilar artery or vertebral artery ) N5 ( ischemi* or ischaemi* or infarct* or thrombo* or emboli* or occlus*)) OR AB ((brain or cerebr* or cerebell* or vertebrobasil* or hemispher* or intracran* or intracerebral or infratentorial or supratentorial or middle cerebral artery or MCA* or anterior circulation or posterior circulation or basilar artery or vertebral artery ) N5 ( ischemi* or ischaemi* or infarct* or thrombo* or emboli* or occlus*))
4. S4 TI (( brain* or cerebr* or cerebell* or intracerebral or intracran* or parenchymal or intraparenchymal or intraventricular or infratentorial or supratentorial or basal gangli* or putaminal or putamen or posterior fossa or hemispher* ) N5 ( haemorrhage* or hemorrhage* or haematoma* or hematoma* or bleed* )) OR AB (( brain* or cerebr* or cerebell* or intracerebral or intracran* or parenchymal or intraparenchymal or intraventricular or infratentorial or supratentorial or basal gangli* or putaminal or putamen or posterior fossa or hemispher* ) N5 ( haemorrhage* or hemorrhage* or haematoma* or hematoma* or bleed* ))
5. S5 S1 OR S2 OR S3 OR S4
6. S6 (MH "Deglutition") OR (MH "Gagging")
7. S7 (MH "Deglutition Disorders")
8. S8 TI ( (swallow* or deglutit* or dysphag*) N3 (disturbance* or disorder* or difficult* or dysfunction* or impair* or condition* or abnormal* or damage* or injur*) ) OR AB ( (swallow* or deglutit* or dysphag*) N3 (disturbance* or disorder* or difficult* or dysfunction* or impair* or condition* or abnormal* or damage* or injur*) )
9. S9 TI ((swallow* or deglutit* or dysphag*) N3 (scale* or screen* or checklist* or assess* or exam* or identif* or recogni* or evaluat* or diagnos* or detect* or hazard or risk or test)) OR AB ((swallow* or deglutit* or dysphag*) N3 (scale* or screen* or checklist* or assess* or exam* or identif* or recogni* or evaluat* or diagnos* or detect* or hazard or risk or test))
10. S10 S6 OR S7 OR S8 OR S9
11. S11 MH Random Assignment or MH Single-blind Studies or MH Double-blind Studies or MH Triple-blind Studies or MH Crossover design or MH Factorial Design
12. S12 TI ("multicentre study" or "multicenter study" or "multi-centre study" or "multi-center study") or AB ("multicentre study" or "multicenter study" or "multi-centre study" or "multi-center study") or SU ("multicentre study" or "multicenter study" or "multi-centre study" or "multi-center study")
13. S13 TI random* or AB random*
14. S14 AB "latin square" or TI "latin square"
15. S15 TI (crossover or cross-over) or AB (crossover or cross-over) or SU (crossover or cross-over)
16. S16 MH Placebos
17. S17 TI ( ((singl* or doubl* or trebl* or tripl*) N3 (blind* or mask*)) ) OR AB ( ((singl* or doubl* or trebl* or tripl*) N3 (blind* or mask*)) )
18. S18 TI Placebo* or AB Placebo* or SU Placebo*
19. S19 MH Clinical Trials
20. S20 TI (Clinical AND Trial) or AB (Clinical AND Trial) or SU (Clinical AND Trial)
21. S21 S11 OR S12 OR S13 OR S14 OR S15 OR S16 OR S17 OR S18 OR S19 OR S20
22. S22 S5 AND S10 AND S21
