# Supplementary material for: Patient Reported Outcome Measures in Dysphagia Research Following Stroke: A Scoping Review and Qualitative Analysis
Source: Dysphagia. 2022 Apr 25;38(1):181–90. doi: 10.1007/s00455-022-10448-y (PMC9873730; doi:10.1007/s00455-022-10448-y)
Supplement: Supplementary file 2 — Supplementary file2 (DOCX 28 kb) [file 455_2022_10448_MOESM2_ESM.docx]

**Supplementary Material: List of 110 RCTs included in Analysis**

1. Arreola, V.; Alvarez-Berdugo, D.; Rofes, L.; Ortega, O.; Muriana, D.; Clavé, P. Clinical and biomechanical effects of transcutaneous electrical stimulation on chronic post-stroke oropharyngeal dysphagia: Results at one year follow up of a randomized controlled trial European Stroke Journal 2019;4():58-592019
2. Arreola, V.; Alvarez-Berdugo, D.; Rofes, L.; Palomera, E.; Tomsen, N.; Ortega, O.; Muriana, D.; Palomeras, E.; Clavé, P. Transcutaneous electrical stimulation improves the swallow safety and reduces the need of fluid thickening in patients with chronic post-stroke oropharyngeal dysphagia European Stroke Journal 2018;3(1):51-52 2018
3. Arreola, V.; Rofes, L.; Vilardell, N.; Tomsen, N.; Alvarez-Berdugo, D.; Ortega, O.; Muriana, D.; Cobo, M.; Clave, P. Therapeutic effect of transcutaneous electrical stimulation on chronic post-stroke oropharyngeal dysphagia: A randomized controlled trial with two stimulation intensities Dysphagia 2018;33(4):519-520 2018
4. Bai J, Li B, Wang Z, Gao W, Wang L. The role of different needling manipulation in adjusting swallow period obstacle of dysphagia after stroke. Zhongguo Zhenjiu 2007; 27(1):35-7.
5. Balashova, I. N.; Vanchakova, N. P.; Afanasiev, V. V.; Barantzevich, E. R.; Pugacheva, E. L.; Golikov, K. V. Diagnosis and treatment of neurogenic dysphagia after acute ischemic stroke Zhurnal Nevrologii i Psihiatrii imeni S.S. Korsakova 2018;118(12):64-69 2018
6. Bath PM, Scutt P, Love J, Clavé P, Cohen D, Dziewas R, et al. Pharyngeal electrical stimulation for treatment of dysphagia in subacute stroke: a randomized controlled trial. Stroke 2016; 47:1562-70.
7. Benfield, J. K.; Everton, L. F.; Bath, P. M.; England, T. J. Dysphagia therapy with surface electromyographic biofeedback: A feasibility randomised controlled trial in acute stroke Dysphagia 2019;34(5):747 2019
8. Bucyana, E.; Dhanalakshmi, R.; Ganesh, S. Influence of swallowing exercises, thermal tactile stimulation and neuromuscular electrical stimulation in treatment of patients with dysphagia caused by stroke Indian Journal of Public Health Research and Development 2019;10(6):683-686 2019
9. Cabib, C.; Nascimento, W.; Rofes, L.; Arreola, V.; Tomsen, N.; Mundet, L.; Palomeras, E.; Michou, E.; Clavé, P.; Ortega, O. Short-term neurophysiological effects of sensory pathway neurorehabilitation strategies on chronic poststroke oropharyngeal dysphagia Neurogastroenterology and Motility 2020;32(9): 2020
10. Cabib, C.; Nascimento, W.; Rofes, L.; Arreola, V.; Tomsen, N.; Mundet, L.; Michou, E.; Clavé, P.; Ortega, O. Stimulation of the swallowing sensory pathway enhances global motor cortical excitability in post-stroke patients with chronic oropharyngeal dysphagia 2020;35(1):1452020
11. Carnaby G, Hankey GJ, Pizzi J. Behavioural intervention for dysphagia in acute stroke: a randomised controlled trial. Lancet Neurology 2006; 5:31-7.
12. Carnaby, G. D.; LaGorio, L.; Silliman, S.; Crary, M. Exercise-based swallowing intervention (McNeill Dysphagia Therapy) with adjunctive NMES to treat dysphagia post-stroke: A double-blind placebo-controlled trial Journal of oral rehabilitation 2020;47(4):501-510 2020
13. Chan S, Or K, Sun W, Ng K, Lo S, Lee Y. Therapeutic effects of acupuncture for neurogenic dysphagia - a randomized controlled trial. Journal of Traditional Chinese Medicine 2012; 32(1):25-30.
14. Chen L, Fang J, Ma R, Froym R, Gu X, Li J, et al. Acupuncture for acute stroke: study protocol for a multicenter, randomized, controlled trial. Trials 2014; 15: 214.
15. Chen L, Fang J, Ma R, Gu X, Chen L, Li J, et al. Additional effects of acupuncture on early comprehensive rehabilitation in patients with mild to moderate acute ischemic stroke: a multicenter randomized controlled trial. BMC Complementary and Alternative Medicine 2016; 16: 226.
16. Chen, X. Q.; Zhu, M. Y.; Zou, Y. C.; Wu, Z. H.; Peng, Y. X.; Huang, F. Effect of "Tongyuan" acupuncture treatment on success rate of extubation in stroke patients undergoing tracheotomy. Acupuncture research 2019;44(9):663-667 2019
17. Cheng, I. K. Y.; Yiu, E. M. L.; Chan, K. M. K. Inter-individual variability in the responses to rTMS as a treatment for chronic post-stroke dysphagia 2019;34(5):748 2019
18. Cui, F.; Yin, Q.; Wu, C.; Shen, M.; Zhang, Y.; Ma, C.; Zhang, H.; Shen, F. Capsaicin combined with ice stimulation improves swallowing function in patients with dysphagia after stroke: A randomised controlled trial Journal of oral rehabilitation 2020;47(10):1297-1303 2020
19. Du J, Yang F, Liu L, Hu J, Cai B, Liu W, et al. Repetitive transcranial magnetic stimulation for rehabilitation of poststroke dysphagia: a randomized, double-blind clinical trial. Clinical Neurophysiology 2016; 127: 2907-13.
20. Dziewas, R.; Stellato, R.; van der Tweel, I.; Walther, E.; Werner, C. J.; Braun, T.; Citerio, G.; Jandl, M.; Friedrichs, M.; Nötzel, K.; Vosko, M. R.; Mistry, S.; Hamdy, S.; McGowan, S.; Warnecke, T.; Zwittag, P.; Bath, P. M.; Aroyo, I.; Bucka, C.; Kerz, T.; Köstenberger, M.; Marschner-Preuth, N.; Niesen, W. D.; Pfausler, B.Pharyngeal electrical stimulation for early decannulation in tracheotomised patients with neurogenic dysphagia after stroke (PHAST-TRAC): a prospective, single-blinded, randomised trial. The Lancet Neurology 2018;17(10):849-859 2018
21. Dziewas, R.; Stellato, R.; Van Der Tweel, I.; Walther, E.; Werner, C.; Braun, T.; Citerio, G.; Jandl, M.; Friedrichs, M.; Nötzel, K.; Vosko, M.; Mistry, S.; Hamdy, S.; McGowan, S.; Warnecke, T.; Zwittag, P.; Bath, P. Pharyngeal electrical stimulation for early decannulation in tracheotomised stroke patients with neurogenic dysphagia (phast-trac): A prospective randomised single blinded interventional study -secondary outcomes. European Stroke Journal 2018;3(1):587 2018
22. Everton, L.; Michou, E.; Hamdy, S.; Bath, P. Effects of pharyngeal electrical stimulation on swallow timings, clearance and safety: Adhoc analysis from the Swallowing Treatment using Electrical Pharyngeal Stimulation (STEPS) Trial International Journal of Stroke 2019;14(4 SUPPL):472019
23. Feng X, Hao W, Ding Z, Sui Q, Guo H, Fu J. Clinical study on tongyan spray for post-stroke dysphagia patients: a randomized controlled trial. Chinese Journal of Integrative Medicine 2012; 18(5):345-9.
24. Guo, Y. H.; Sun, Z. R.; Cai, S.; Jin, Z.; Wei, Q. S.; Jiang, F.; Wang, M. Z.; Yin, H. N. Fire-needle therapy for deglutition disorders in post-stroke pseudobulbar palsy: a randomized controlled trial Journal of Acupuncture and Tuina Science Dec 2018;16(6):375-381 2018 Dec
25. Hägglund, P.; Hägg, M.; Levring Jäghagen, E.; Larsson, B.; Wester, P. Oral neuromuscular training in patients with dysphagia after stroke: a prospective, randomized, open-label study with blinded evaluators BMC Neurology 2020;20(1): 2020
26. Hamdy, S.; Dziewas, R.; Van Der Tweel, I.; Bath, P. Pharyngeal electrical stimulation for early decannulation in tracheotomised stroke patients with dysphagia (phast-trac): A randomised, single-blind, pivotal, superiority trial Dysphagia 2019;34(3):455 2019
27. Han JC. An observation on the therapeutic effect of acupuncture for bulbar palsy after acute stroke. Henan Journal of Practical Nervous Diseases 2004; 7(3):81-2.
28. Hao, W. J.; Li, B. J.; Wu, X. L.; Chen, Z. B.; Zhang, X.; Yao, H.; Zhang, W. Y.; Feng, X. G. Effect and Safety of Tongyan Sprayon Hyoid Motion in Patients with Dysphagia after Ischemic Stroke Chinese Journal of Integrative Medicine 2020;(): 2020
29. Hendy, R. M.; Elerian, A. E.; Emara, T. H Effect of transcutaneous electrical nerve stimulation and conventional therapy in post-stroke dysphagic patients: a randomized controlled trial Bioscience Research Jan-Mar 2019;16(1):11-16 2019 Jan-Mar
30. Heo SY, Kim KM. Immediate effects of kinesio taping on the movement of the hyoid bone and epiglottis during swallowing by stroke patients with dysphagia. Journal of Physical Therapy Science 2015; 27:3355–7.
31. Huang Z, Huang F, Yan HX, Min Y, Gao Y, Tan BD, et al. Dysphagia after stroke treated with acupuncture or electric stimulation: a randomized controlled trial. Zhongguo Zhen Jiu 2010; 30(12):969-73.
32. Huang, Y. Q.; Ma, W.; Shen, W. D. Efficacy evaluation of acupuncture plus rehabilitation training for post-stroke deglutition disorders of qi-deficiency blood stasis pattern Journal of Acupuncture and Tuina Science Oct 2020;18(5):367-373 2020 Oct
33. Hwang, N. K.; Kim, H. H.; Shim, J. M.; Park, J. S. Tongue stretching exercises improve tongue motility and oromotor function in patients with dysphagia after stroke: A preliminary randomized controlled trial Archives of oral biology 2019;108():104521 2019
34. Jang, K. W.; Lee, S. J.; Kim, S. B.; Lee, K. W.; Lee, J. H.; Park, J. G. Effects of mechanical inspiration and expiration exercise on velopharyngeal incompetence in subacute stroke patients Journal of rehabilitation medicine 2019;51(2):97-102 2019
35. Jayasekeran V, Singh S, Tyrrell P, Michou E, Jefferson S, Mistry S, et al. Adjunctive functional pharyngeal electrical stimulation reverses swallowing disability after brain lesions. Gastroenterology 2010; 138(5):1737-46.
36. Jeon, Y. H.; Cho, K. H.; Park, S. J. Effects of neuromuscular electrical stimulation (NMES) plus upper cervical spine mobilization on forward head posture and swallowing function in stroke patients with dysphagia Brain Sciences 2020;10(8):1-10 2020
37. Jia H, Zhang Y. Treatment of 40 cases of post-apoplectic dysphagia by acupuncture plus rehabilitation exercise. Journal of Acupuncture and Tuina Science 2006; 4(6):336-8.
38. Jin, H. P.; Li, X. L.; Ye, Q. J.; Wang, Y. Effect of electrical stimulation with bilateral scalp acupuncture on time parameters in video fluoroscopic swallowing study and cortical excitability in patients with dysphagia after cortical stroke Zhen ci yan jiu = Acupuncture research 2020;45(6):473-479 2020
39. Kang J, Park R, Lee S, Kim J, Yoon S, Jung K. The effect of bedside exercise program on stroke patients with dysphagia. Annals of Rehabilitation Medicine 2012; 26:512-20.
40. Khedr EM, Abo-Elfetoh N, Rothwell JC. Treatment of post-stroke dysphagia with repetitive transcranial magnetic stimulation. Acta Neurologica Scandinavica 2009;119(3):155-61
41. Khedr EM, Abo-Elfetoh N. Therapeutic role of rTMS on recovery of dysphagia in patients with lateral medullary syndrome and brainstem infarction. Journal of Neurology Neurosurgery and Psychiatry 2010; 81:495-9.
42. Kim L, Chun MH, Kim BR, Lee SJ. Effect of repetitive transcranial magnetic stimulation on patients with brain injury and dysphagia. Annals of Rehabilitation Medicine 2011; 35:765-71.
43. Kim, H. H.; Park, J. S. Efficacy of modified chin tuck against resistance exercise using hand-free device for dysphagia in stroke survivors: A randomised controlled trial Journal of oral rehabilitation 2019;46(11):1042-1046 2019
44. Kim, Y. K.; Lee, K. Y.; Lee, S. H. Efficacy of a 4-Week Swallowing Rehabilitation Program Combined With Pyriform Sinus Ballooning in Patients With Post-stroke Dysphagia Annals of Rehabilitation Medicine-Arm Aug 2018;42(4):542-550
45. Konecny, P.; Elfmark, M. Electrical stimulation of hyoid muscles in post-stroke dysphagia Biomedical papers of the Medical Faculty of the University Palacky, Olomouc, Czechoslovakia 2018;162(1):40-42 2018
46. Krajczy, E.; Krajczy, M.; Luniewski, J.; Bogacz, K.; Szczegielniak, J. Assessment of the effects of dysphagia therapy in patients in the early post-stroke period: A randomised controlled trial Neurologia i Neurochirurgia Polska 2019;53(6):428-434 2019
47. Kumar S, Wagner CW, Frayne C, Zhu L, Selim M, Feng W, et al. Noninvasive brain stimulation may improve stroke-related dysphagia: a pilot study. Stroke 2011; 42(4):1035-40.
48. Lee JS, Chui PY, Ma HM, Auyeung TW, Kng C, Law T, et al. Does low dose angiotensin converting enzyme inhibitor prevent pneumonia in older people with neurologic dysphagia - a randomized placebo-controlled trial. Journal of the American Medical Directors Association 2015; 16(8):702-7.
49. Lee KW, Kim SB, Lee JH, Lee SJ, Ri JW, Park JG. The effect of early neuromuscular electrical stimulation therapy in acute/ subacute ischemic stroke patients with dysphagia. Annals of Rehabilitation Medicine 2014; 38(2):153-9.
50. Li L, Shi J, Yin J, Qiao B, Li Y, Huang R. Study of transcutaneous neuromuscular electrical stimulation (VitalStim) therapy for post-stroke dysphagia. European Journal of Physical and Rehabilitation Medicine 2014; Jul: 23.
51. Li, L.; Li, Y.; Wu, X.; Wang, G.; Yi, X.; Zhao, Y.; Guo, M.; Pan, M.; Tang, C. The Value of Adding Transcutaneous Neuromuscular Electrical Stimulation (VitalStim) to Traditional Therapy for Poststroke Dysphagia: A Randomized Controlled Trial Topics in Geriatric Rehabilitation 2018;34(3):200-206 2018
52. Li, Y.; Feng, X.; Yang, Z.; Wang, T.; Qin, H.; Lu, Y.; Li, S. Mechanism of deglutition stage acupuncture for treating deglutition disorder after stroke Pakistan Journal of Pharmaceutical Sciences 2020;33(1):307-3152020
53. Li, Y.; Li, S.; Feng, X.; Qin, H.; Jin, X.; Niu, L. A Clinical Study on the Treatment of Dysphagia after Stroke with Five Acupuncture Combined with Taste Stimulation Basic and Clinical Pharmacology and Toxicology 2020;127(SUPPL 1):140-141 2020
54. Lim KB, Lee HJ, Lim SS, Choi YI. Neuromuscular electrical and thermal-tactile stimulation for dysphagia caused by stroke: a randomized controlled trial. Journal of Rehabilitation Medicine 2009; 41(3):174-8.
55. Liu L. Acupuncture treatment of bulbar palsy - a report of 54 cases. Journal of Traditional Chinese Medicine 2000; 20(1):30-2.
56. Liu Y. Treatment of pseudobulbar paralysis by scalp acupuncture and sublingual needling. Journal of Traditional Chinese Medicine 2004; 24(1):26-7.
57. Liu, X. P.; Chen, F. Y.; Chu, J. M.; Bao, Y. H. Therapeutic observation of Gao's nape acupuncture plus swallowing training for pharyngeal deglutition disorder after stroke Journal of Acupuncture and Tuina Science Feb 2019;17(1):37-43 2019
58. Meng, P.; Zhang, S.; Wang, Q.; Wang, P.; Han, C.; Gao, J.; Yue, S. The effect of surface neuromuscular electrical stimulation on patients with post-stroke dysphagia Journal of Back and Musculoskeletal Rehabilitation 2018;31(2):363-370 2018
59. Moon, J. H.; Hahm, S. C.; Won, Y. S.; Cho, H. Y. The effects of tongue pressure strength and accuracy training on tongue pressure strength, swallowing function, and quality of life in subacute stroke patients with dysphagia: a preliminary randomized clinical trial International journal of rehabilitation research. Internationale Zeitschrift fur Rehabilitationsforschung. Revue internationale de recherches de readaptation 2018;41(3):204-210 2018
60. Moon, J. H.; Heo, S. J.; Jung, J. H. Effects of orofacial muscles exercise program on swallowing function and satisfaction in sub-acute stroke patients with dysphagia Medico-Legal Update 2019;19(1):623-628 2019
61. Oh, D. H.; Park, J. S.; Kim, H. J.; Chang, M. Y.; Hwang, N. K. The effect of neuromuscular electrical stimulation with different electrode positions on swallowing in stroke patients with oropharyngeal dysphagia: A randomized trial Journal of Back and Musculoskeletal Rehabilitation 2020;33(4):637-644 2020
62. Park E, Kim MS, Chang WH, Oh SM, Kim YK, Lee A, Kim Y. Effects of bilateral repetitive transcranial magnetic stimulation on post stroke dysphagia. Brain Stimulation 2017; 10:75-82.
63. Park J, Kim Y, Oh J, Lee H. Effortful swallowing training combined with electrical stimulation in post-stroke dysphagia: a randomized controlled study. Dysphagia 2012; 27:521-7.
64. Park J, Oh J, Lee J, Yeo J, Ryu KH. The effect of 5Hz highfrequency rTMS over contralesional pharyngeal motor cortex in post-stroke oropharyngeal dysphagia: a randomized controlled study. Neurogastroenterology and Motility 2013; 25:324–e250.
65. Park JS, Oh DH, Chang MY, Kim KM. Effects of expiratory muscle strength training on oropharyngeal dysphagia in subacute stroke patients: a randomised controlled trial. Journal of Oral Rehabilitation 2016; 43:364-72.
66. Park, H. S.; Oh, D. H.; Yoon, T.; Park, J. S. Effect of effortful swallowing training on tongue strength and oropharyngeal swallowing function in stroke patients with dysphagia: a double-blind, randomized controlled trial International Journal of Language & Communication Disorders 2019;54(3):479-484 2019
67. Park, J. S.; An, D. H.; Kam, K. Y.; Yoon, T.; Kim, T.; Chang, M. Y. Effects of resistive jaw opening exercise in stroke patients with dysphagia: A double- blind, randomized controlled study Journal of Back and Musculoskeletal Rehabilitation 2020;33(3):507-513 2020
68. Park, J. S.; An, D. H.; Oh, D. H.; Chang, M. Y. Effect of chin tuck against resistance exercise on patients with dysphagia following stroke: A randomized pilot study NeuroRehabilitation 2018;42(2):191-197 2018
69. Park, J. S.; Lee, G.; Jung, Y. J. Effects of game-based chin tuck against resistance exercise vs head-lift exercise in patients with dysphagia after stroke: An assessor-blind, randomized controlled trial Journal of rehabilitation medicine 2019;51(10):749-754 2019
70. Perez I, Smithard DG, Davies H, Kalra L. Pharmacological treatment of dysphagia in stroke. Dysphagia 1998; 13:12-6.
71. Pingue, V.; Priori, A.; Malovini, A.; Pistarini, C. Dual transcranial direct current stimulation for poststroke dysphagia: A randomized controlled trial Neurorehabilitation and Neural Repair 2018;32(6-7):635-644 2018
72. Ploumis, A.; Papadopoulou, S. L.; Theodorou, S. J.; Exarchakos, G.; Givissis, P.; Beris, A. Cervical isometric exercises improve dysphagia and cervical spine malalignment following stroke with hemiparesis: a randomized controlled trial European journal of physical and rehabilitation medicine 2018;54(6):845-852 2018
73. Power ML, Fraser DH, Hobson A, Singh S, Tyrell P, Nicholson DA, et al. Evaluating oral stimulation as a treatment for dysphagia after stroke. Dysphagia 2006; 21(1):49-55.
74. Qin, L.; Zhang, X. P.; Yang, X. C.; Cui, C. H.; Shi, J.; Jia, C. S.Deep acupuncture of Lianquan (CV23) and Yifeng (TE17) in combination with conventional acupuncture of other acupoints is superior to swallowing rehabilitation training in improving post-stroke dysphagia in apoplexy patients. Acupuncture research 2019;44(2):144-147 2019
75. Rogus-Pulia, N. M.; Yee, J.; Rogus-Pulia, N. M.; Yee, J.; Knigge, M. A.; Thibeault, S.; Young, B.; Sattin, J.; Borders, J.; Thibeault, S.; Gangnon, R. Effects of device-facilitated lingual strengthening therapy on swallowing-related outcomes post-stroke: A pilot study Dysphagia 2019;34(3):434 2019
76. Sawan, S. A. E.; Reda, A. M.; Kamel, A. H.; Ali, M. A. M. Transcranial direct current stimulation (tDCS): its effect on improving dysphagia in stroke patients Egyptian Journal of Neurology, Psychiatry and Neurosurgery 2020;56(1): 2020
77. Shigematsu T, Fujishima I, Ohno K. Transcranial direct current stimulation improves swallowing function in stroke patients. Neurorehabilitation and Neural Repair 2013; 27(4):363-9.
78. Simonelli, M.; Ruoppolo, G.; Iosa, M.; Morone, G.; Fusco, A.; Grasso, M. G.; Gallo, A.; Paolucci, S. A stimulus for eating. the use of neuromuscular transcutaneous electrical stimulation in patients affected by severe dysphagia after subacute stroke: A pilot randomized controlled trial NeuroRehabilitation 2019;44(1):103-110 2019
79. Smaoui, S.; Steele, C.; Peladeau-Pigeon, M.; Richardson, D. Tongue pressure training protocol for swallowing impairment post-stroke International Journal of Stroke 2019;14(3):30-31 2019
80. Song QL. Swallowing and ingesting training and nursing in patients with swallowing disorders after stroke. Chinese Journal of Clinical Rehabilitation 2004;8(19):3722-3
81. Sproson, L.; Pownall, S.; Enderby, P.; Freeman, J. Combined electrical stimulation and exercise for swallow rehabilitation post-stroke: a pilot randomized control trial International Journal of Language & Communication Disorders 2018;53(2):405-417 2018
82. Sun, D.; Xu, W.; Chen, N.; Li, S. M.; Fu, T. Clinical Effectiveness of Intradermal Needle-embedding Therapy for Swallowing Function in Stroke Patients with Dysphagia. Acupuncture research 2018;43(2):118-122 2018
83. Suntrup-Krueger, S.; Ringmaier, C.; Muhle, P.; Wollbrink, A.; Kemmling, A.; Hanning, U.; Claus, I.; Warnecke, T.; Teismann, I.; Pantev, C.; Dziewas, R. Randomized trial of transcranial direct current stimulation for poststroke dysphagia Annals of neurology 2018;83(2):328-340 2018
84. Tarameshlu, M.; Ansari, N. N.; Ghelichi, L.; Jalaei, S. The effect of repetitive transcranial magnetic stimulation combined with traditional dysphagia therapy on poststroke dysphagia: a pilot double-blinded randomized-controlled trial International journal of rehabilitation research. Internationale Zeitschrift fur Rehabilitationsforschung. Revue internationale de recherches de readaptation 2019;42(2):133-138 2019
85. Terre R, Mearin F. A randomized controlled study of neuromuscular electrical stimulation in oropharyngeal dysphagia secondary to acquired brain injury. European Journal of Neurology 2015; 22(4):687-e44.
86. Ünlüer, N. Ö; Temuçin, Ç M.; Demir, N.; Serel Arslan, S.; Karaduman, A. A. Effects of Low-Frequency Repetitive Transcranial Magnetic Stimulation on Swallowing Function and Quality of Life of Post-stroke Patients Dysphagia 2019;34(3):360-371 2019
87. Ünlüer,; Temuçin, C.; Demir, N.; Arslan, S.; Karaduman, A. The effects of 1Hz low frequency transcranial magnetic stimulation on swallowing function in post-stroke dysphagia Dysphagia 2018;33(4):492 2018
88. Vasant D, Michou E, Tyrrell P, Jayasekeran V, Mistry S, O'Leary N, et al. Pharyngeal electrical stimulation (PES) In dysphagia post-acute stroke: a double-blind, randomised trial. Gut 2014; 63(1):A31.
89. Vasant, DH, Michou E, O'Leary N, Vail A, Mistry S, Hamdy S, et al. Pharyngeal electrical stimulation in dysphagia poststroke: a prospective, randomized single-blinded interventional study. Neurorehabilitation and Neural Repair 2016;30(9):866-75
90. Vose, A.; Humbert, I. A.; Marcus, A. Kinematic visual biofeedback improves accuracy of swallowing maneuver training and accuracy of clinician cues during training in post-stroke patients with dysphagia 2019;34(3):460 2019
91. Wang, W.; Jia, Y.; Cai, H.; Wang, W.; Sun, C. Functional patch combined with surface electromyographic biofeedback for post-stroke dysphagia Chinese Journal of Tissue Engineering Research 2020;24(29):4697-4701 2020
92. Wang, Z. Y.; Chen, J. M.; Lin, Z. K.; Ni, G. X. Transcranial direct current stimulation improves the swallowing function in patients with cricopharyngeal muscle dysfunction following a brainstem stroke Neurological Sciences 2020;41(3):569-574 2020
93. Wang, Z.; Wu, L.; Fang, Q.; Shen, M.; Zhang, L.; Liu, X. Effects of capsaicin on swallowing function in stroke patients with dysphagia: A randomized controlled trial Journal of Stroke and Cerebrovascular Diseases 2019;28(6):1744-1751 2019
94. Warusevitane AB, Karunatilake DS, Sim J, Lally F, Roffe C. Safety and effect of metoclopramide to prevent pneumonia in patients with stroke fed via nasogastric tubes trial. Stroke 2015;46:454-60
95. Wei LL. Effect of shuiti acupoint injection with stellate ganglion block on swallow dysfunction after stroke. Chinese Journal of Clinical Rehabilitation 2005; 9(9):106-7.
96. Wilkinson, G.; Sasegbon, A.; Smith, C. J.; Rothwell, J.; Bath, P. M.; Hamdy, S. An Exploration of the Application of Noninvasive Cerebellar Stimulation in the Neuro-rehabilitation of Dysphagia after Stroke (EXCITES) Protocol Journal of Stroke and Cerebrovascular Diseases 2020;29(3): 2020
97. Wu, W. B.; Fan, D. F.; Zheng, C.; Que, B. F.; Lian, Q. Q.; Qiu, R.; Chen, Y. G.; Pan, L. Y.; Zhang, Y. Relieving throat and opening orifice acupuncture therapy for the post-stroke dysphagia World Journal of Acupuncture - Moxibustion 2019;29(1):37-41 2019
98. Xia W, Zheng C, Lei Q, Tang Z, Hua Q, Zhang Y, et al. Treatment of post-stroke dysphagia by vitalStim therapy coupled with conventional swallowing training. Journal of Huazhong University of Science and Technology - Medical Sciences 2011; 31(1):73-6.
99. Xia W, Zheng C, Zhu, Tang Z. Does the addition of specific acupuncture to standard swallowing training improve outcomes in patients with dysphagia after stroke? A randomized controlled trial. Clinical Rehabilitation 2016; 30(3):237-46.
100. Xiao, H. Y.; Han, L. P.; Xu, J. Efficacy of acupuncture treatment on dysphagia staging after acute ischemic stroke Acta Medica Mediterranea 2019;35(2):683-687 2019
101. Xing, B. F.; Zhou, X.; Deng, X. Q. Effect of "Tongdu Tiaoshen" needling combined with swallowing training on dysphagia, cerebral blood flow and serum BDNF and NGF levels in ischemic stroke patients. Acupuncture research 2019;44(7):506-5112019
102. Xu, Z. J.; Xiang, L.; Liu, X. Observation on mind-refreshing and orifice-opening needling method plus swallowing disorder therapeutic apparatus for deglutition disorder of stroke patients in the convalescent stage Journal of Acupuncture and Tuina Science Jun 2018;16(3):156-160 2018 Jun
103. Yuan ZH, Huang LL, Chen ZL. Coagulant and enteral nutrition agents in the rehabilitation of deglutition disorders for patients with acute stroke. Chinese Journal of Clinical Rehabilitation 2003; 7(28):3834-5.
104. Yuan, Y.; Cai, X. H.; Chen, F.; Chen, D. X.; Gao, Y.; Liu, Z. Z.; Ling, M. X.; Xu, P. Clinical trials of acupuncture treatment of post-stroke dysphagia by deep acupuncture of Tiantu (CV22) in combination with swallowing rehabilitation training. Acupuncture research 2019;44(1):47-50 2019
105. Zhang, C.; Zheng, X.; Lu, R.; Yun, W.; Yun, H.; Zhou, X. Repetitive transcranial magnetic stimulation in combination with neuromuscular electrical stimulation for treatment of post-stroke dysphagia Journal of International Medical Research 2019;47(2):662-672 2019
106. Zhang, R.; Ju, X. M. Clinical improvement of nursing intervention in swallowing dysfunction of elderly stroke patients Biomedical Research (India) 2018;29(6):1099-1102 2018
107. Zhang, W.; Yang, H.; Yao, T. Effect of bundles of care on rehabilitation effect of stroke patients with dysphagia Acta Medica Mediterranea 2020;36(6):3601-3606 2020
108. Zhao, L.; Liu, L.; Zhang, C. S.; Zeng, L.; Zhao, J.; Wang, L.; Jing, X.; Wang, K.; Li, B. Effect of He's Santong Needling Method on Dysphagia after Stroke: A Study Protocol for a Prospective Randomized Controlled Pilot Trial Evidence-Based Complementary and Alternative Medicine 2018;2018(): 2018
109. Zhao, W.; Ju, C.; Wang, D.; Shen, H. Clinical observation of effects of ultrashort wave therapy combined with acupuncture and rehabilitation training in the treatment of patients with dysphagia after stroke Journal of Neurorestoratology 2019;7(3):136-142 2019
110. Zheng L, Li Y, Liu Y. The individualized rehabilitation interventions for dysphagia: a multidisciplinary case control study of acute stroke patients. International Journal of Clinical and Experimental Medicine 2014; 7(10):3789-94.
