# Supplementary material for: Patient Reported Outcome Measures in Dysphagia Research Following Stroke: A Scoping Review and Qualitative Analysis
Source: Dysphagia. 2022 Apr 25;38(1):181–90. doi: 10.1007/s00455-022-10448-y (PMC9873730; doi:10.1007/s00455-022-10448-y)
Supplement: Supplementary file 3 — Supplementary file3 (DOCX 33 kb) [file 455_2022_10448_MOESM3_ESM.docx]

**Supplementary Material: SWAL-QOL mapped to ICF and COMET Taxonomy**

| item | icf concept | icf component | icf chapter | icf category | comet core area | comet outcome domain |
| --- | --- | --- | --- | --- | --- | --- |
| Dealing with my swallowing problem is very difficult | Swallowing  Burden | Body Functions (b)  Body Functions (b) | 5 Functions of the digestive, metabolic and endocrine systems  2 General tasks and demands | B510 Ingestion functions  D230 Carrying out daily routine | Life Impact | Emotional functioning/ wellbeing |
| My swallowing problem is a major distraction in my life | Swallowing  Burden | Body Functions (b)  Body Functions (b) | 5 Functions of the digestive, metabolic and endocrine systems  2 General tasks and demands | B510 Ingestion functions  D230 Carrying out daily routine | Life Impact | Emotional functioning/ wellbeing |
| Most days, I don’t care if I eat or not | Eating  Apathy  Appetite | Activities and Participation (d)  Unable to categorise  Body Functions (b) | 5 Self-care  1 Mental functions | D550 Eating  B130 Energy and drive functions | Physiological/ Clinical | Metabolism and nutrition outcomes |
| It takes me longer to eat than other people | Eating  Time Taken | Activities and Participation (d)  Body Functions (b) | 5 Self-care  2 General tasks and demands | D550 Eating  D230 Carrying out daily routine | Life Impact | Physical functioning |
| I’m rarely hungry anymore | Appetite | Body Functions (b) | 1 Mental functions | B130 Energy and drive functions | Physiological/ Clinical | Metabolism and nutrition outcomes |
| It takes me forever to eat a meal | Eating  Time Taken | Activities and Participation (d)  Body Functions (b) | 5 Self-care  2 General tasks and demands | D550 Eating  D230 Carrying out daily routine | Life Impact | Physical functioning |
| I don’t enjoy eating anymore | Eating  Pleasure | Activities and Participation (d)  Activities and Participation (d) | 5 Self-care  9 Community, social and civic life | D550 Eating  D920 Recreation and leisure | Life Impact | Emotional functioning/ wellbeing |
| How often in the last month, have you experienced choking with eating food? | Choking  Eating | Body Functions (b)  Activities and Participation (d) | 4 Functions of the cardiovascular, haematological, immunological and respiratory systems  5 Self-care | B450 Additional functions of the respiratory system  D550 Eating | Physiological/ clinical | Respiratory, thoracic and mediastinal outcomes |
| How often in the last month, have you experienced choking when you take liquids? | Choking  Drinking | Body Functions (b)  Activities and Participation (d) | 4 Functions of the cardiovascular, haematological, immunological and respiratory systems  5 Self-care | B450 Additional functions of the respiratory system  D560 Drinking | Physiological/ clinical | Respiratory, thoracic and mediastinal outcomes |
| How often in the last month, have you experienced having thick saliva or phlegm? | Saliva management | Body Functions (b) | 5 Functions of the digestive, metabolic and endocrine systems | B510 Ingestion functions | Physiological/ clinical | Gastrointestinal outcomes |
| How often in the last month, have you experienced gagging? | Gagging | Body Functions (b) | 4 Functions of the cardiovascular, haematological, immunological and respiratory systems | B450 Additional functions of the respiratory system | Physiological/ clinical | Respiratory, thoracic and mediastinal outcomes |
| How often in the last month, have you experienced drooling? | Saliva management | Body Functions (b) | 5 Functions of the digestive, metabolic and endocrine systems | B510 Ingestion functions | Physiological/ clinical | Gastrointestinal outcomes |
| How often in the last month, have you experienced problems chewing? | Chewing | Body Functions (b) | 5 Functions of the digestive, metabolic and endocrine systems | B510 Ingestion functions | Physiological/ clinical | Gastrointestinal outcomes |
| How often in the last month, have you experienced having excess saliva and phlegm? | Saliva management | Body Functions (b) | 5 Functions of the digestive, metabolic and endocrine systems | B510 Ingestion functions | Physiological/ clinical | Gastrointestinal outcomes |
| How often in the last month, have you experienced having to clear your throat? | Management of phlegm/secretions | Body Functions (b) | 5 Functions of the digestive, metabolic and endocrine systems | B510 Ingestion functions | Physiological/ clinical | Gastrointestinal outcomes |
| How often in the last month, have you experienced food sticking in your throat? | Food sticking | Body Functions (b) | 5 Functions of the digestive, metabolic and endocrine systems | B280 Sensation of pain | Physiological/ clinical | Gastrointestinal outcomes |
| How often in the last month, have you experienced food sticking in your mouth? | Food sticking | Body Functions (b) | 2 Sensory functions and pain | B280 Sensation of pain | Physiological/ clinical | Gastrointestinal outcomes |
| How often in the last month, have you experienced food or liquid dribbling out of your mouth? | Oral control | Body Functions (b) | 5 Functions of the digestive, metabolic and endocrine systems | B510 Ingestion functions | Physiological/ clinical | Gastrointestinal outcomes |
| How often in the last month, have you experienced food or liquid coming out your nose? | Nasal regurgitation | Body functions (b) | 5 Functions of the digestive, metabolic and endocrine systems | B510 Ingestion functions | Physiological/ clinical | Gastrointestinal outcomes |
| How often in the last month, have you experienced coughing food or liquid out of your mouth when it gets stuck? | Expectoration of food/liquid | Body Functions (b) | 4 Functions of the cardiovascular, haematological, immunological and respiratory systems | B450 Additional functions of the respiratory system | Physiological/ clinical | Respiratory, thoracic and mediastinal outcomes |
| Figuring out what I can and can’t eat is a problem for me. | Navigating swallowing difficulties | Activities and participation (d) | 1 Learning and applying knowledge | D177 Making decisions | Life Impact | Emotional functioning/ wellbeing |
| It is difficult to find foods that I both like and can eat | Limited choices | Activities and participation (d) | 1 Learning and applying knowledge | D177 Making decisions | Life Impact | Social functioning |
| I fear I may start choking when I eat food | Choking | Body Functions (b) | 4 Functions of the cardiovascular, haematological, immunological and respiratory systems | B450 Additional functions of the respiratory system | Life Impact | Emotional functioning/ wellbeing |
| I worry about getting pneumonia | Worry/Anxiety  Pneumonia | Activities and participation (d)  Body Functions (b) | 2 General tasks and demands  4 Functions of the cardiovascular, haematological, immunological and respiratory systems | D240 Handling stress and other physiological demands  B450 Additional functions of the respiratory system | Life Impact | Emotional functioning/ wellbeing |
| I am afraid of choking when I drink liquids | Worry/Anxiety  Choking  Drinking | Activities and participation (d)  Body Functions (b)  Activities and Participation (d) | 2 General tasks and demands  4 Functions of the cardiovascular, haematological, immunological and respiratory systems  5 Self-care | D240 Handling stress and other physiological demands  B450 Additional functions of the respiratory system  D560 Drinking | Life Impact | Emotional functioning/ wellbeing |
| I never know when I am going to choke | Unpredictability  Choking | Unable to categorise  Body Functions (b) | 4 Functions of the cardiovascular, haematological, immunological and respiratory systems | B450 Additional functions of the respiratory system | Life Impact | Emotional functioning/ wellbeing |
| My swallow problem depresses me | Swallowing  Impact on mental health | Body Functions (b)  Unable to categorise | 5 Functions of the digestive, metabolic and endocrine system | B510 Ingestion functions | Life Impact | Emotional functioning/ wellbeing |
| Having to be so careful when I eat and drink annoys me | Caution  Frustration | Unable to categorise  Unable to categorise |  |  | Life Impact | Emotional functioning/ wellbeing |
| I’ve been discouraged by my swallowing problem | Swallowing  Motivation | Body Functions (b)  Body Functions (b) | 5 Functions of the digestive, metabolic and endocrine system  1 Mental functions | B510 Ingestion functions  B130 Energy and drive functions | Life Impact | Emotional functioning/ wellbeing |
| My swallowing problem frustrates me | Swallowing  Frustration | Body Functions (b)  Unable to categorise | 5 Functions of the digestive, metabolic and endocrine system | B510 Ingestion functions | Life Impact | Emotional functioning/ wellbeing |
| I get impatient dealing with my swallowing problem | Swallowing  Frustration | Body Functions (b)  Unable to categorise | 5 Functions of the digestive, metabolic and endocrine system | B510 Ingestion functions | Life Impact | Emotional functioning/ wellbeing |
| I do not go out because of my swallowing problems | Swallowing  Social Impact | Body Functions (b)  Activities and Participation (d) | 5 Functions of the digestive, metabolic and endocrine system  9 Community, social and civic life | B510 Ingestion functions  D920 Recreation and leisure | Life Impact | Social functioning |
| My swallowing problem makes it hard to have a social life | Swallowing  Social Impact | Body Functions (b)  Activities and Participation (d) | 5 Functions of the digestive, metabolic and endocrine system  9 Community, social and civic life | B510 Ingestion functions  D920 Recreation and leisure | Life Impact | Social functioning |
| My usual work or leisure activities have changed because of my swallowing problem | Swallowing  Change in normal routine | Body Functions (b)  Activities and Participation (d) | 5 Functions of the digestive, metabolic and endocrine system  2 General tasks and demands | B510 Ingestion functions  D230 Carrying out daily routine | Life Impact | Role functioning |
| Social gatherings (like holidays or get-togethers) are not enjoyable because of my swallowing problem | Social gatherings | Activities and Participation (d) | 9 Community, social and civic life | D920 Recreation and leisure | Life Impact | Social functioning |
| My role with family and friends has changed because of my swallowing problem | Swallowing  Familiar Roles | Body Functions (b)  Activities and Participation (d) | 5 Functions of the digestive, metabolic and endocrine systems  7 Interpersonal interactions and relationships | B510 Ingestion functions  D770 Intimate relationships | Life Impact | Role functioning |

**Supplementary Material: EAT-10 mapped to ICF and COMET Taxonomy**

| item | icf concept | icf component | icf chapter | icf category | comet core area | comet outcome domain |
| --- | --- | --- | --- | --- | --- | --- |
| My swallowing problem has caused me to lose weight | Swallowing  Lose Weight | Body Functions (b)  Body Functions (b) | 5 Functions of the digestive, metabolic and endocrine systems  5 Functions of the digestive, metabolic and endocrine systems | B510 Ingestion functions  B530 Weight maintenance functions | Physiological/ Clinical | Metabolism and nutrition outcomes |
| My swallowing interferes with my ability to go out for meals | Swallowing  Go out for meals | Body Functions (b)  Activities and Participation (d) | 5 Functions of the digestive, metabolic and endocrine systems  9 Community, social and civic life | B510 Ingestion functions  D920 Recreation and leisure | Life Impact | Social functioning |
| Swallowing liquid takes extra effort | Swallowing  Drinking  Effort | Body Functions (b)  Activities and Participation (d)  Body Functions (b) | 5 Functions of the digestive, metabolic and endocrine systems  5 Self-care  2 General tasks and demands | B510 Ingestion functions  D560 Drinking  D230 Carrying out daily routine | Life Impact | Physical functioning |
| Swallowing solids takes extra effort | Swallowing  Eating  Effort | Body Functions (b)  Activities and Participation (d)  Body Functions (b) | 5 Functions of the digestive, metabolic and endocrine systems  5 Self-care  2 General tasks and demands | B510 Ingestion functions  D550 Eating  D230 Carrying out daily routine | Life Impact | Physical functioning |
| Swallowing pills takes extra effort | Swallowing  Medication  Effort | Body Functions (b)  Activities and Participation (d)  Body Functions (b) | 5 Functions of the digestive, metabolic and endocrine systems  5 Self-care  2 General tasks and demands | B510 Ingestion functions  D570 Looking after one’s health  D230 Carrying out daily routine | Life Impact | Physical functioning |
| Swallowing is painful | Swallowing  Painful | Body Functions (b)  Body Functions (b) | 5 Functions of the digestive, metabolic and endocrine systems  2 Sensory functions and pain | B510 Ingestion functions  B280 Sensation of pain | Physiological/ Clinical | General outcomes |
| The pleasure of eating is affected by my swallowing | Eating  Pleasure  Swallowing | Activities and Participation (d)  Activities and Participation (d)  Body Functions (b) | 5 Self-care  9 Community, social and civic life  5 Functions of the digestive, metabolic and endocrine systems | D550 Eating  D920 Recreation and leisure  B510 Ingestion functions | Life Impact | Emotional functioning/ wellbeing |
| When I swallow, food sticks in throat | Food sticking  Swallowing  Throat | Body Functions (b)  Body Functions (b)  Body Structures (s) | 2 Sensory functions and pain  5 Functions of the digestive, metabolic and endocrine systems  3 Structures involved in voice and speech | B280 Sensation of pain  B510 Ingestion functions  S330 Structure of pharynx | Physiological/ clinical | Gastrointestinal outcomes |
| I cough when I eat | Cough  Eating | Body Functions (b)  Activities and Participation (d) | 4 Functions of the cardiovascular, haematological, immunological and respiratory systems  5 Self-care | B450 Additional functions of the respiratory system  D550 Eating | Physiological/ clinical | Respiratory, thoracic and mediastinal outcomes |
| Swallowing is stressful | Swallowing  Stressful | Body Functions (b)  Activities and Participation (d) | 5 Functions of the digestive, metabolic and endocrine systems  2 General tasks and demands | B510 Ingestion functions  D240 Handling stress and other physiological demands | Life Impact | Emotional functioning/ wellbeing |

**Supplementary Material: Frequency of ICF categories**

| **ICF CATEGORY** | **TOTAL NO. OF TIMES IDENTIFIED/ FREQUENCY** | **NO. OF TIMES IDENTIFIED IN SWAL-QOL/ FREQUENCY** | **NO. OF TIMES IDENTIFIED IN EAT-10/ FREQUENCY** |
| --- | --- | --- | --- |
| b510 – Ingestion functions | n=27, 35% | n=18, 34% | n=9, 36% |
| b450 – Additional functions of the respiratory system | n=10, 12% | n=9, 17% | n=1, 4% |
| d550 – Eating | n=8, 10% | n=5, 9% | n=3, 12% |
| d230 – Carrying out daily routine | n=8, 10% | n=5, 9% | n=3, 12% |
| d920 – Recreation and leisure | n=6, 8% | n=4, 8% | n=2, 8% |
| b280 – Sensation of pain | n=4, 5% | n=2, 4% | n=2, 8% |
| b130 – Energy and drive functions | n=3, 4% | n=3, 6% | 0 |
| d560 – Drinking | n=3, 4% | n=2, 4% | n=1, 4% |
| d240 – Handling stress and other physiological demands | n=3, 4% | n=2, 4% | n=1, 4% |
| d177 – Making decisions | n=2, 3% | n=2, 4% | 0 |
| b530 – Weight maintenance functions | n=1, 1% | 0 | n=1, 4% |
| d570 – Looking after one’s health | n=1, 1% | 0 | n=1, 4% |
| d770 – Intimate relationships | n=1, 1% | n=1, 2% | 0 |
| s330 – Structure of pharynx | n=1, 1% | 0 | n=1, 4% |

**Supplementary Material: Frequency of COMET Taxonomy outcome domains**

| **COMET TAXONOMY OUTCOME DOMAIN** | **TOTAL NO. OF TIMES IDENTIFIED/ FREQUENCY** | **NO. OF TIMES IDENTIFIED IN SWAL-QOL/ FREQUENCY** | **NO. OF TIMES IDENTIFIED IN EAT-10/ FREQUENCY** |
| --- | --- | --- | --- |
| Emotional functioning/wellbeing | n=15, 32% | n=13, 35% | n=2, 20% |
| Gastrointestinal outcomes | n=10, 21% | n=9, 24% | n=1, 10% |
| Respiratory, thoracic and mediastinal outcomes | n=6, 13% | n=5, 14% | n=1, 10% |
| Social functioning | n=5, 11% | n=4, 11% | n=1, 10% |
| Physical functioning | n=5, 11% | n=2, 5% | n=3, 30% |
| Metabolism and nutrition outcomes | n=3, 6% | n=2, 5% | n=1, 10% |
| Role functioning | n=2, 4% | n=2, 5% | 0 |
| General outcomes | n=1, 2% | 0 | n=1, 10% |
